# Hepatitis B Virus Infection Is Associated with a Higher Risk of Liver Metastasis in Gastric Cancer

**DOI:** 10.3390/curroncol33030179

**Published:** 2026-03-21

**Authors:** Songting Zhu, Mengmeng Jiang, Yanyan Chen, Yongfeng Ding, Haiyong Wang, Lisong Teng

**Affiliations:** 1 Department of Surgical Oncology, The First Affiliated Hospital, School of Medicine, Zhejiang University, Hangzhou 310003, China; 2 Department of Medical Oncology, The First Affiliated Hospital, School of Medicine, Zhejiang University, Hangzhou 310003, China

**Keywords:** gastric cancer, liver metastasis, hepatitis B virus, prognosis

## Abstract

Background: Hepatitis B virus infection has been linked to liver cancer and may influence metastasis in other malignancies, but its role in gastric cancer liver metastasis (GCLM) is unclear. Methods: We retrospectively analyzed 776 gastric cancer patients with HBV testing. HBV infection was defined as HBsAg+ (chronic HBV, CHB) or HBsAg− with HBcAb/HBeAb+ (occult HBV, OHB). Among the 776 patients, 300 (38.6%) were classified as HBV+. The association between HBV infection and GCLM was evaluated, and propensity score matching (PSM) was performed to adjust for age and gender. Furthermore, the impact of HBV infection on overall survival (OS) was analyzed. Results: GCLM occurred in 19.5% of patients. HBV+ patients had a higher GCLM prevalence than HBV− patients (25.3% vs. 15.8%; *p* = 0.001), persisting after PSM (25.3% vs. 15.3%; *p* = 0.002). HBV infection was an independent risk factor for GCLM (OR = 2.563, *p* < 0.001). Both OHB and CHB groups showed significantly higher GCLM rates than HBV− patients in univariate and multivariate analyses. However, OS did not differ between groups (*p* = 0.737). Conclusion: HBV infection significantly increases the risk of liver metastasis in gastric cancer. Enhanced surveillance for liver metastasis is warranted in these patients.

## Simple Summary

Hepatitis B virus (HBV) is not only associated with primary liver cancer but also increases the risk of other malignancies, and may be linked to an increased risk of liver metastasis in colorectal cancer. However, the relationship between HBV infection and liver metastasis in gastric cancer (GCLM) remains unclear. This retrospective study focuses on the relationship between HBV infection and liver metastasis in gastric cancer. This study shows that hepatitis B virus infection increases the risk of liver metastasis in gastric cancer patients. Patients with HBV infection should receive careful monitoring for liver metastasis to improve clinical management and outcomes.

## 1. Introduction

Gastric cancer (GC) remains a major global health burden, ranking among the top five cancers worldwide in terms of both incidence and mortality [1]. Despite substantial advances in diagnostic techniques and therapeutic strategies, the prognosis for GC patients remains poor, with five-year survival rates particularly low in those with advanced disease [2]. One of the most critical factors contributing to high mortality in GC is the occurrence of distant metastasis. Among potential metastatic sites, the liver is especially vulnerable due to its unique anatomical and physiological characteristics, including its dual blood supply from the portal vein and hepatic artery and the presence of a rich sinusoidal microvasculature [3]. Approximately 2.0% to 9.9% of gastric cancer patients present with liver metastasis at the time of diagnosis, known as synchronous gastric cancer liver metastasis. Additionally, up to 37% of gastric cancer patients develop liver metastasis after curative gastrectomy [4]. The presence of liver metastasis not only limits surgical and systemic treatment options but also dramatically reduces overall survival, highlighting the urgent need to identify high-risk patients.

The cellular and molecular mechanisms of tumor distant metastasis are complex, involving both intrinsic properties of cancer cells and interactions with the target organ microenvironment. One well-known theory is the “seed and soil” hypothesis, which suggests that ability of tumor cells (“seeds”) to establish metastatic colonies depends on the receptive conditions of the distant organ microenvironment (“soil”) [5,6]. Most studies on gastric cancer metastasis focus on the invasive properties of the gastric cancer cells themselves [7,8], the role of the hepatic microenvironment in supporting metastatic colonization remains poorly understood. Factors such as local immune status, hepatic stellate cell activation, cytokine profiles, and extracellular matrix composition may all influence the likelihood of liver metastasis. Identifying these contributing factors is essential for developing targeted interventions and improving patient outcomes.

Hepatitis B virus (HBV) infection is highly prevalent in China [9], and studies have confirmed that HBV plays a significant role in the development of liver-related diseases, particularly primary hepatocellular carcinoma [10,11]. Several population-based studies have reported associations between chronic HBV infection and an increased risk of extrahepatic cancers, including cervical cancer [12], esophagus cancer, pancreatic cancer, non-Hodgkin lymphoma, leukemia [13] and gastric cancer [12,13,14,15]. Beyond its established role in primary liver malignancies, emerging evidence indicates that HBV may also modulate the hepatic microenvironment in ways that influence the risk and progression of secondary liver tumors. A study in colorectal cancer has suggested that HBV infection can increase the likelihood of liver metastasis [16]. However, despite the biological plausibility and epidemiological clues, the specific role of HBV infection in gastric cancer liver metastasis (GCLM) remains largely unexplored. Therefore, we conducted this retrospective cohort study to systematically investigate the association between HBV infection and the occurrence of liver metastasis in gastric cancer patients.

## 2. Materials and Methods

### 2.1. Patients

**Figure 1:**
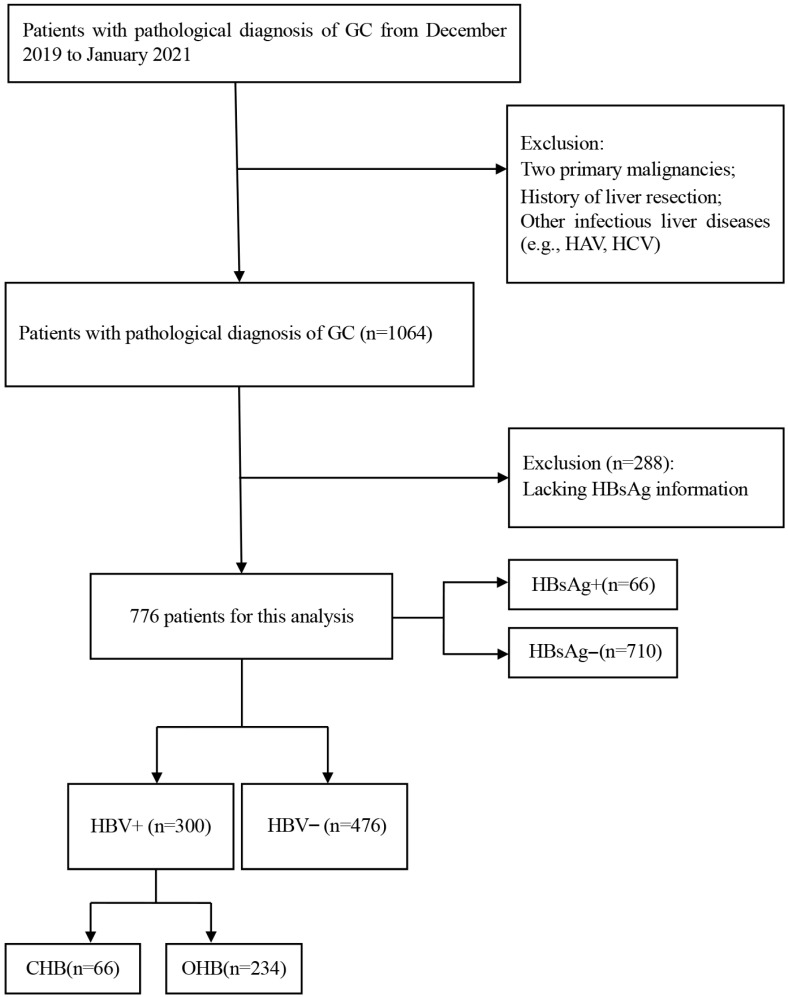
The flow chart of the study.

This study included patients diagnosed with gastric cancer at the First Affiliated Hospital of Zhejiang University School of Medicine between December 2019 and January 2021. Eligible patients were those with histopathologically confirmed gastric adenocarcinoma who had no other primary tumors, no history of liver surgery, and no other infectious liver diseases. A total of 1064 patients were initially enrolled. After excluding 288 patients with missing HBsAg data, 776 patients were included in the final analysis (Figure 1).

### 2.2. Clinical Data

Clinical information collected in this study was divided into four major categories. The first category included demographic characteristics and clinical history, specifically patient age, gender, and the presence of liver cirrhosis. The second category consisted of tumor-related pathological features, including tumor location, tumor diameter, histological differentiation, and TNM stage. The third category comprised laboratory indicators, which included HBV-related serological markers (HBsAg, HBsAb, HBeAg, HBeAb, HBcAb), commonly used tumor biomarkers (CA199, CEA, and AFP), and routine biochemical parameters (ALT, AST, GGT, ALP, ALB, AG, TBIL, DBIL, IBIL, LDH, HB, and PLT). The fourth category encompassed tumor treatment modalities, including details on systemic therapy, surgical procedures (e.g., radical surgery with D2 lymphadenectomy, palliative surgery), and other relevant interventions. All laboratory parameters included in the analysis were obtained within 30 days before the pathological diagnosis of GC.

### 2.3. HBV Infection Criteria

Based on the patients’ HBVM, cases were initially categorized into two groups: HBV-infected (HBV+) and HBV-uninfected (HBV−). The HBV+ group includes patients with chronic hepatitis B (CHB) who are HBsAg positive, and those with occult HBV infection (OHB), which includes patients who are HBsAg negative but HBcAb or HBeAb positive. To further clarify the composition of the study population, we also defined the following subgroups based on HBsAg status. The HBsAg+ group was a subgroup of the HBV+ population consisting of patients with detectable HBsAg, indicating current HBV infection. The HBsAg− group included all patients who tested negative for HBsAg, regardless of their HBcAb or HBeAb status. For HBsAg-negative individuals, the presence of HBcAb and/or HBeAb was considered as serological evidence of previous HBV exposure and these patients were categorized into the occult HBV infection-related group in the present study. According to the institutional clinical practice guideli1nes for HBV management, patients newly diagnosed with HBV infection at our center underwent HBV DNA testing and received guideline-directed antiviral therapy. Patients with known HBV infection were routinely followed up at specialized hepatology outpatient clinics.

### 2.4. Definition of Liver Metastasis

The diagnosis of liver metastasis was primarily established through regular follow-up imaging examinations. Patients typically underwent contrast-enhanced computed tomography (CT) or magnetic resonance imaging (MRI) every 3 to 6 months after the initial gastric cancer treatment. For the vast majority of patients, the diagnosis was based on typical radiological findings of liver metastases. All imaging results were independently reviewed by at least two experienced radiologists who were blinded to the patients’ clinical outcomes to ensure consistency. In a small subset of cases where imaging findings were indeterminate or when clinically indicated, the diagnosis was further confirmed by histopathological examination of ultrasound- or CT-guided percutaneous liver biopsy specimens. Metastasis occurring within six months or less after the pathological diagnosis of GC is classified as synchronous metastasis, while metastasis occurring more than six months later is classified as metachronous metastasis.

### 2.5. Follow-Up

All patients were regularly followed up after initial treatment according to institutional guidelines. Follow-up visits were scheduled every 3–6 months for the first 2 years, every 6–12 months for the next 3 years, and annually thereafter. Each follow-up included physical examination, laboratory tests, and imaging examinations. Contrast-enhanced CT of the chest, abdomen, and pelvis was the primary modality for detecting recurrence and metastasis. Abdominal ultrasonography was routinely performed to evaluate the liver. MRI or biopsy was performed when necessary to confirm suspicious lesions. Overall survival (OS) was defined as the time from diagnosis to death or last follow-up. Follow-ups were conducted via outpatient visit or telephone interviews.

### 2.6. Propensity Score Matching

To minimize potential baseline differences between the groups, propensity score matching (PSM) was performed. Propensity scores were estimated using a logistic regression model in which HBV infection status (HBV+ vs. HBV−) was treated as the dependent variable, while age and gender were included as covariates. The calculated propensity score represented the estimated probability of each patient being classified into the HBV+ group based on these variables. Patients in the HBV+ group were then matched to those in the HBV− group at a 1:1 ratio using the nearest-neighbor matching method without replacement. A caliper width equal to 0.2 standard deviations of the logit-transformed propensity score was applied to restrict the matching process. After matching, the comparability between the two groups was evaluated by calculating standardized differences for all covariates. A standardized difference value of <0.1 was considered to indicate acceptable balance between the matched groups.

### 2.7. Statistical Analysis

The Kolmogorov–Smirnov test was used to assess the normality of continuous variables. For variables with a normal distribution, data were presented as mean ± standard deviation, and comparisons between two groups were performed using Student’s *t*-test. For variables with a non-normal distribution, data were expressed as median with interquartile range, and comparisons between two groups were conducted using the Mann–Whitney U test or Kruskal–Wallis H test. Categorical variables were assessed using the chi-square test. In addition, multivariate logistic regression analyses were performed both before and after PSM to evaluate the potential association between HBV infection and gastric cancer liver metastasis (GCLM). Survival analysis was performed using the Kaplan–Meier method, and the log-rank test was used to analyze the effect of HBV infection on the prognosis of GC. Statistical analysis was conducted using SPSS 26 (IBM). Statistical significance was defined as a two-tailed *p*-value < 0.05.

## 3. Results

### 3.1. Baseline Characteristics and Balancing Groups by PSM

The baseline characteristics are shown in Table S1. A total of 151 of the 776 gastric cancer patients (19.5%) developed liver metastases, including 109 patients with synchronous lesions and 42 patients with metachronous lesions during follow-up. Among the 776 gastric cancer patients, 300 (38.6%) were in the HBV+ group. There were no significant differences between the HBV+ and HBV− groups in terms of gender, primary tumor location, tumor size, differentiation, and staging. To minimize the impact of confounding factors, we balanced the differences between the two groups using PSM. We performed a 1:1 matching based on gender and age, resulting in a final dataset of 600 patients. The baseline characteristics of the two groups after PSM are presented in Table 1, showing no significant differences between the matched groups.

### 3.2. Association Between HBV and GCLM

The incidence of liver metastasis in the HBV+ group was 25.3% (76/300), significantly higher than the 15.8% (75/476) observed in the HBV− group (*p* = 0.001, Table 1). This difference remained significant after PSM as well (25.3% vs. 15.3%, *p* = 0.002, Table 1).

To further explore the correlation between HBV infection and liver metastasis, we conducted a univariate analysis (Table S2). The results showed that univariate analysis revealed that gender, tumor size, tumor grade, T stage, N stage, systemic therapy, HBV infection, AFP, CEA, CA19-9, ALB, ALT, AST, ALP, GGT, HB, and PLT were significantly associated with the presence of liver metastasis (all *p* < 0.05). All variables with *p* < 0.05 in univariate analysis were subsequently entered into a multivariate logistic regression model (Table 2). After adjusting for potential confounders, HBV infection remained an independent risk factor for the occurrence of liver metastasis (OR = 2.563, 95% CI: 1.647–3.990, *p* < 0.001), and this association persisted even after PSM (OR = 2.900, 95% CI: 1.727–4.871, *p* < 0.001). These findings suggest a significant correlation between HBV infection and liver metastasis in gastric cancer, with HBV+ patients having a risk of liver metastasis that is twice that of HBV− patients.

### 3.3. Association Between HBsAg and GCLM

In our cohort, the positive rate of HBsAg was 8.5% (66/776). Based on the HBsAg status, patients were categorized into HBsAg+ and HBsAg− groups, and we compared the baseline characteristics between the two groups (Table 3). The results indicated that patients in the HBsAg+ group exhibited a trend towards younger age (63.5 vs. 69.0, *p* < 0.001), while the other characteristics remained consistent. The incidence of liver metastasis was higher in the HBsAg+ group compared to the HBsAg− group (24.2% vs. 19.0%, *p* = 0.305), although this difference did not reach statistical significance.

### 3.4. Impact of Different HBV Infection Statuses on GCLM

The above analyses demonstrated that patients with HBV infection had a higher incidence of GCLM than those without HBV infection; however, no statistically significant association was observed between HBsAg positivity and liver metastasis, despite a higher liver metastasis rate in the HBsAg+ group compared with the HBsAg− group. Therefore, we further compared the effects of different HBV infection statuses on GCLM. Patients were categorized into three groups: the HBV− group, the OHB group, and the CHB group. Comparisons of clinicopathological characteristics among the three groups revealed no statistically significant differences in gender, tumor location, tumor size, tumor differentiation grade, T stage, or N stage. Patients in the CHB group were younger than those in the HBV− and OHB groups (63.5 vs. 70.0 vs. 69.0, *p* < 0.001, Table 4), and the incidence of liver metastasis was significantly higher in both the OHB and CHB groups than in the HBV-negative group (25.6% vs. 24.2% vs. 15.8%, *p* = 0.004, Table 4).

Further multivariate analysis demonstrated that the risks of liver metastasis in the OHB (OR = 2.328, 95% CI: 1.495–3.623, *p* < 0.001, Table 5) and CHB (OR = 2.687, 95% CI: 1.335–5.406, *p* = 0.006, Table 5) groups were significantly higher than those in the HBV− group, indicating that both OHB and CHB status were independent prognostic factors for liver metastasis.

### 3.5. Subgroup Analysis Stratified by Initial Resectability Status

Of the 776 patients with gastric adenocarcinoma enrolled in this study, 517 (66.6%) were initially diagnosed with resectable disease and underwent curative-intent surgery (including those who received neoadjuvant therapy followed by surgery and those who underwent upfront surgery). The remaining 259 patients (33.4%) were deemed unresectable at diagnosis due to metastatic disease. Among these, 60 patients (23.2%) received palliative surgery, and the other 204 patients (78.8%) received non-surgical palliative treatment. To further elucidate the role of HBV exposure in different clinical contexts, we performed separate analyses stratified by initial resectability status.

In the 517 patients with initially resectable disease, 30 (5.8%) developed liver metastases after curative-intent surgery. Univariate analysis revealed that HBV exposure was associated with an increased risk of postoperative liver metastases (10.9% vs. 2.5%, *p* < 0.001) (Table S3). Multivariate analysis adjusting for clinicopathological factors including age, tumor size, TN stage, systematic treatment, CEA, CA199, GGT and HB confirmed that HBV exposure was an independent predictor of liver metastases in this subgroup (OR = 2.111, 95% CI: 1.145–3.894, *p* = 0.017) (Table 6).

Among the 259 patients with initially unresectable disease, 121 (46.7%) developed liver metastases during the disease course (including both synchronous metastases present at diagnosis and metachronous metastases that developed during follow-up). Univariate analysis showed that HBV exposure was significantly associated with the occurrence of liver metastases in this subgroup (55.1% vs. 41.6%, *p* = 0.035) (Table S4). After adjusting for gender, tumor size, AFP, CEA, ALT, AST, ALP and GGT, multivariate logistic regression confirmed that HBV exposure remained an independent risk factor for liver metastases in initially unresectable patients (OR = 2.640, 95% CI: 1.379–5.056, *p* = 0.003) (Table 7).

### 3.6. Influence of HBV on the Prognosis of Gastric Cancer

**Figure 2:**
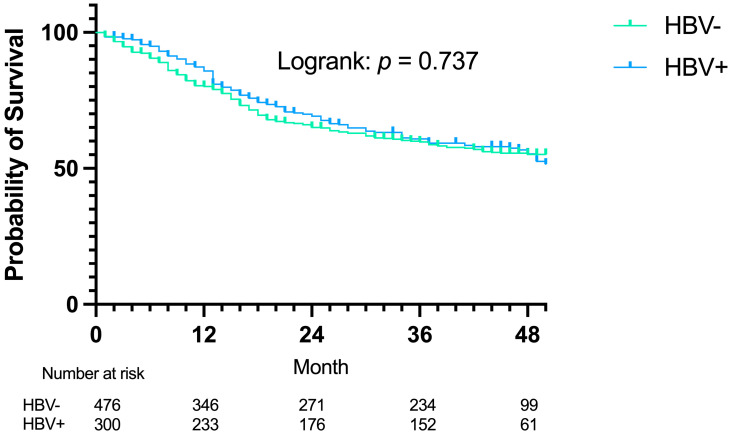
Effect of hepatitis B virus (HBV) infection on the overall survival (OS) of gastric cancer (GC).

We further assessed the prognostic impact of HBV infection in gastric cancer patients. With a median follow-up of 47 months (last follow-up: 31 September 2024), Kaplan–Meier analysis revealed no significant difference in overall survival between HBV-positive and HBV-negative patients (log-rank *p* = 0.737; Figure 2). Subgroup analyses stratified by initial resectability status similarly showed no significant survival difference associated with HBV infection in either the resectable (log-rank *p* = 0.738; Figure S1) or unresectable group (log-rank *p* = 0.355; Figure S2). Collectively, these findings indicate that while HBV infection increases the risk of liver metastasis, it does not significantly influence the overall survival of gastric cancer patients.

## 4. Discussion

Liver metastasis remains a major clinical challenge in gastric cancer (GC), as the liver is the most frequent site of hematogenous dissemination, and once metastasis occurs, therapeutic options are limited with poor prognosis [17]. The liver’s susceptibility to metastasis is largely attributable to its unique anatomical and hemodynamic features: a dual blood supply from the portal vein and hepatic artery exposes it to a high burden of circulating tumor cells, while slow sinusoidal flow facilitates tumor cell adhesion and extravasation. Additionally, structural elements such as fenestrated sinusoidal endothelium and an immune-tolerant hepatic microenvironment create a receptive “soil” that supports metastatic colonization [18,19]. While most studies on tumor metastasis focus on the intrinsic invasive properties of tumor cells, the well-known “seed and soil” hypothesis emphasizes that the compatibility of the target organ microenvironment is equally critical. In the case of GC, the liver provides a favorable niche for circulating tumor cells; however, it remains unclear whether host factors, such as hepatitis B virus (HBV) infection, modulate its susceptibility to metastasis. In this retrospective study, we observed that HBV-infected GC patients exhibited a significantly higher risk of developing liver metastasis, suggesting that HBV may influence the hepatic pre-metastatic niche and promote tumor colonization.

China has a high prevalence of HBV, with reported infection rates in the general population ranging from 5% to 6% [9]. In our cohort, the positivity rate of HBsAg among gastric cancer patients was 8.5%, slightly higher than that of the general population. Previous studies in colorectal cancer have reported an increased incidence of liver metastasis among HBV-infected patients, indicating that HBV infection may influence tumor metastasis by altering the hepatic microenvironment [20,21,22]. These observations provide indirect support for the hypothesis that HBV-related changes in the liver may facilitate metastatic colonization of gastrointestinal tumors. In the present study, HBV infection was significantly associated with an increased risk of liver metastasis in gastric cancer patients, and this association remained robust after propensity score matching and multivariate analysis. Stratified analysis according to initial resectability further demonstrated that this relationship was consistent in both resectable and unresectable groups, suggesting that the influence of HBV on hepatic metastasis may be largely independent of the initial surgical status of the tumor and reinforces the notion that HBV may contribute to creating a liver microenvironment favorable for metastatic colonization. This finding is in line with the concept that HBV infection may contribute to creating a hepatic microenvironment that favors tumor cell implantation and growth. These results underscore the importance of considering HBV status in risk assessment and surveillance strategies for gastric cancer patients, regardless of their initial surgical eligibility. In regions with a high prevalence of HBV infection, such as China, identifying HBV-positive patients may help clinicians recognize individuals at higher risk of hepatic metastasis and potentially guide more intensive surveillance strategies during follow-up.

Although the underlying mechanisms were not directly investigated in this study, several biological pathways may explain the observed association between HBV infection and gastric cancer liver metastasis (GCLM). HBV infection may reshape the hepatic microenvironment, creating a “fertile soil” that facilitates the adhesion, colonization, and growth of metastatic gastric cancer cells. For instance, HBsAg has been reported to stimulate dendritic cells to secrete various cytokines, promoting tumorigenesis in HBV-infected tissues [23], while the HBV-encoded X protein (HBx) is closely associated with tumor progression and metastasis [10,24,25]. Additionally, studies in colorectal cancer have shown that HBV infection increases the expression of chemokines such as MCP-1, CCL20, and CXCL9/10 [26,27], which are closely related to colorectal cancer metastasis [28,29,30]. Notably, these chemokines have also been implicated in the progression and metastasis of GC [31,32,33]. Beyond liver metastasis, epidemiological evidence suggests that HBV infection may also contribute to gastric carcinogenesis. A meta-analysis of ten studies concluded that HBV infection significantly increases the risk of gastric cancer [34]. Interestingly, a cohort in Iran observed that co-infection with HBV and *Helicobacter pylori* markedly increased the risk compared with *H. pylori* infection alone [35]. HBV may act synergistically with *H. pylori* to promote gastric precancerous lesions and malignant progression [36,37,38]. Taken together, these findings suggest that HBV infection may reshape the hepatic microenvironment, thereby facilitating the colonization and growth of metastatic gastric cancer cells.

To further investigate the impact of different hepatitis B infection statuses on liver metastasis of gastric cancer, we conducted a stratified analysis. Firstly, we compared the influence of HBsAg on GCLM. Analysis of baseline characteristics revealed a significant age difference between the HBsAg+ and HBsAg− groups, highlighting a trend of earlier gastric cancer onset in HBsAg+ patients. A large cohort study involving 40,963 cancer patients and 5715 non-cancer patients found that the median age at diagnosis for HBsAg+ cancer patients was significantly lower than that of HBsAg− patients [39]. Although the incidence of liver metastasis was higher in the HBsAg+ group compared to the HBsAg− group, the difference was not statistically significant. Subsequently, we performed a subgroup analysis of the HBV+ group, dividing it into chronic hepatitis B and occult hepatitis B based on HBsAg status. In the subgroup analysis, the risks of GCLM in CHB and OHB group were both significantly higher than HBV− group, indicating HBV infection status affecting the incidence of liver metastasis in gastric cancer. Given that only 66 patients in our cohort were HBsAg+, this may not accurately reflect the relationship between HBsAg and GCLM.

We also evaluated the impact of HBV infection on overall survival (OS) in gastric cancer patients. To our knowledge, there have been few studies focusing on the long-term prognosis of gastric cancer patients in relation to HBV infection. The role of HBV as a prognostic factor in cancer remains controversial, with some studies reporting a protective effect [40,41], and others linking HBsAg positivity to earlier tumor onset and poorer outcomes [39]. In our cohort, no significant difference in OS was observed between HBV+ and HBV− groups. Subgroup analysis according to initial resectability confirmed this finding, with OS remaining similar in both resectable and unresectable groups. This limited effect may be explained by several factors. The proportion of patients with liver metastasis in our cohort was relatively small, which could attenuate the influence of HBV on population-level survival outcomes. Additionally, cancer prognosis is multifactorial, depending on tumor stage, patient comorbidities, and treatment modalities, making it difficult for a single factor to exert a detectable effect on OS. Furthermore, the sample size and follow-up duration in our study may have been insufficient to capture subtle survival differences, suggesting that larger cohorts with extended follow-up are needed to more accurately assess the prognostic impact of HBV.

Several additional limitations should be acknowledged. First, as a single-center retrospective study, the sample size in our cohort was relatively limited, particularly for HBsAg-positive patients, which may introduce residual confounding and affect the statistical robustness of subgroup analyses. Second, the follow-up duration was relatively short, which may have limited our ability to detect potential differences in overall survival between HBV-positive and HBV-negative patients. Third, we did not systematically measure HBV DNA levels or evaluate the potential impact of antiviral therapy, as this information is frequently unavailable or ambiguous in our cohort. Finally, as a single-center study conducted in a high HBV prevalence region, the generalizability of our findings to other populations or ethnicities may be limited. Future large-scale, prospective studies with more complete viral and treatment data, as well as longer follow-up, are needed to validate these results and clarify underlying mechanisms.

## 5. Conclusions

The risk of liver metastasis is significantly increased in gastric cancer patients with HBV infection. Within the follow-up period, no association between HBV infection and overall survival was observed. These findings highlight the importance of identifying HBV infection as a potential risk factor for gastric cancer liver metastasis and may inform risk-adapted surveillance and management strategies.

## Figures and Tables

**Table 1:** Baseline characteristics of gastric cancer (GC) in hepatitis B virus-infected (HBV+) and hepatitis B virus-uninfected (HBV−) groups.

Characteristic	Before PSM			After PSM		
	HBV+ (*n* = 300)	HBV− (*n* = 476)	*p*	HBV+ (*n* = 300)	HBV− (*n* = 300)	*p*
Gender			0.368			0.928
Male	214 (71.3)	325 (68.3)		214 (71.3)	213 (71.0)	
Female	86 (28.7)	151 (31.7)		86 (28.7)	87 (29.0)	
Age, y	68.0 (60.0, 74.0)	70.0 (62.0, 75.0)	0.040	68.0 (60.0, 74.0)	68.0 (61.0, 74.0)	0.525
Primary GC						
Location			0.296			0.508
Cardia	40 (13.3)	51 (10.7)		40 (13.3)	36 (12.0)	
Body	141 (47.0)	212 (44.5)		141 (47.0)	131 (43.7)	
Pylorus	119 (39.7)	213 (44.8)		119 (39.7)	133 (44.3)	
Tumor size, cm	3.6 (3.0, 5.1)	3.6 (2.6, 5.1)	0.986	3.6 (3.0, 5.1)	3.6 (2.6, 5.1)	0.616
Grade			0.110			0.190
Poor	196 (65.3)	337 (70.8)		196 (65.3)	211 (70.3)	
Moderate/Well	104 (34.7)	139 (29.2)		104 (34.7)	89 (29.7)	
T stage			0.978			0.705
T1–T2	76 (25.3)	121 (25.4)		76 (25.3)	72 (24.0)	
T3–T4	224 (75.7)	355 (74.6)		224 (74.7)	228 (76.0)	
N stage			0.283			0.186
N0–N1	134 (44.7)	194 (40.8)		134 (44.7)	118 (39.3)	
N2–N3	166 (55.3)	282 (59.2)		166 (55.3)	182 (60.7)	
Systematic treatment			0.229			0.324
No	63 (21.0)	126 (26.5)		61 (20.3)	71 (23.7)	
Yes	237 (79.0)	350 (73.5)		239 (79.7)	229 (76.3)	
Liver metastasis			0.001			0.002
No	224 (74.7)	401 (84.2)		224 (74.7)	254 (84.7)	
Yes	76 (25.3)	75 (15.8)		76 (25.3)	46 (15.3)	

GC: gastric cancer; HBV+: hepatitis B virus infection; HBV−: hepatitis B virus uninfected; PSM: propensity score matching; y, years.

**Table 2:** Effect of hepatitis B virus (HBV) infection on liver metastasis in gastric cancer (GCLM) before and after propensity score matching (multivariate analysis).

	Before PSM (*n* = 776)			After PSM (*n* = 600)		
	OR	95%CI	*p*	OR	95%CI	*p*
Gender			0.012			
Male	1	1		/	/	
Female	0.507	0.298–0.862		/	/	
Primary GC						
Tumor size, cm	1.221	1.100–1.355	<0.001	1.210	1.077–1.359	0.001
Grade			0.287			0.768
Poor	1	1		1	1	
Moderate/Well	0.737	0.421–1.292		1.093	0.605–1.976	
T stage			0.037			0.021
T1–T2	1	1		1	1	
T3–T4	3.899	1.082–14.043		6.909	1.344–35.516	
N stage			0.001			<0.001
N0–N1	1	1		1	1	
N2–N3	2.919	1.579–5.398		5.099	2.350–11.062	
Systematic treatment			0.031			
No	1	1		/	/	
Yes	2.363	1.081–5.165		/	/	
HBV			<0.001			<0.001
HBV−	1	1		1	1	
HBV+	2.563	1.647–3.990		2.900	1.727–4.871	
AFP	1.002	1.000–1.003	0.010	1.002	1.000–1.003	0.024
CEA	1.000	1.000–1.000	0.847	1.000	1.000–1.000	0.513
CA199	1.000	1.000–1.001	0.057	1.000	1.000–1.000	0.063
ALB	0.992	0.940–1.048	0.778	0.978	0.918–1.043	0.503
ALT	0.992	0.973–1.011	0.413	0.986	0.964–1.008	0.044
AST	0.994	0.973–1.015	0.573	1.021	0.981–1.063	0.215
ALP	1.000	1.000–1.001	0.958	1.000	0.999–1.000	0.537
GGT	1.014	1.007–1.020	<0.001	1.021	1.012–1.030	<0.001
HB	0.991	0.981–1.001	0.098	0.992	0.981–1.004	0.195
PLT	1.001	0.998–1.002	0.984	0.999	0.992–1.002	0.999

GC: gastric cancer; HBV+: hepatitis B virus infection; HBV−: hepatitis B virus uninfected; PSM: propensity score matching; AFP, alpha-fetoprotein; ALB, albumin; ALP, alkaline phosphatase; ALT, alanine aminotransferase; AST, aspartate aminotransferase; CA199, carbohydrate antigen 199; CEA, carcinoembryonic antigen; GGT, γ-glutamyltransferase; HB, hemoglobin; HBV, hepatitis B virus; PLT, platelet.

**Table 3:** Effect of HBsAg status on liver metastasis in gastric cancer (GCLM).

Characteristic	HBsAg+ (*n* = 66)	HBsAg− (*n* = 710)	*p*
Gender			0.427
Male	43 (65.2)	496 (69.9)	
Female	23 (34.8)	214 (30.1)	
Age, y	63.5 (57.8, 70.0)	69.0 (62.0, 75.0)	<0.001
Primary GC			
Location			0.051
Cardia	4 (6.1)	87 (12.3)	
Body	39 (59.1)	314 (44.2)	
Pylorus	23 (34.8)	309 (43.5)	
Tumor size, cm	3.6 (3.0, 5.1)	3.6 (2.8, 5.1)	0.747
Grade			0.644
Poor	47 (71.2)	486 (68.5)	
Moderate/Well	19 (28.8)	224 (31.5)	
T stage			0.507
T1–T2	19 (28.8)	178 (25.1)	
T3–T4	47 (71.2)	532 (74.9)	
N stage			0.419
N0–N1	31 (47.0)	297 (41.8)	
N2–N3	35 (53.0)	413 (58.2)	
Liver metastasis			0.305
No	50 (75.8)	575 (81.0)	
Yes	16 (24.2)	135 (19.0)	

GC: gastric cancer; y, years.

**Table 4:** Subgroup analysis of liver metastasis in gastric cancer (GCLM) based on HBV infection statuses (univariate analysis).

Characteristic	HBV− (*n* = 476)	OHB (*n* = 234)	CHB (*n* = 66)	*p*
Gender				0.311
Male	325 (68.3)	171 (73.1)	43 (65.2)	
Female	151 (31.7)	63 (26.9)	23 (34.8)	
Age, y	70.0 (62.0, 75.0)	69.0 (61.0, 75.0)	63.5 (57.8, 70.0)	<0.001
Primary GC				
Location				0.051
Cardia	51 (10.7)	36 (15.4)	4 (6.1)	
Body	212 (44.5)	102 (43.6)	39 (59.1)	
Pylorus	213 (44.8)	96 (41.0)	23 (34.8)	
Tumor size, cm	3.6 (3.0, 5.1)	3.6 (3.0, 5.1)	3.6 (2.5, 5.1)	0.943
Grade				0.141
Poor	337 (70.8)	149 (63.7)	47 (71.2)	
Moderate/Well	139 (29.2)	85 (36.3)	19 (28.8)	
T stage				0.766
T1–T2	121 (25.4)	103 (24.4)	19 (28.8)	
T3–T4	355 (74.6)	131 (75.6)	47 (71.2)	
N stage				0.512
N0–N1	194 (40.8)	31 (44.0)	31 (47.0)	
N2–N3	282 (59.2)	35 (56.0)	35 (53.0)	
Liver metastasis				0.004
No	401 (84.2)	174 (74.4)	50 (75.8)	
Yes	75 (15.8)	60 (25.6)	16 (24.2)	

GC: gastric cancer; HBV−: hepatitis B virus uninfected; CHB: chronic hepatitis B; OHB: occult HBV infection; y, years.

**Table 5:** Subgroup analysis of liver metastasis in gastric cancer (GCLM) based on HBV infection statuses (multivariate analysis).

	Liver Metastasis		
	OR	95% CI	*p*
Age	1.018	0.997–1.039	0.095
AFP	1.001	1.000–1.002	0.028
CEA	1.000	1.000–1.000	0.683
CA199	1.000	1.000–1.000	0.001
ALB	0.965	0.915–1.018	0.191
ALT	0.987	0.968–1.007	0.211
AST	0.998	0.977–1.019	0.834
ALP	1.000	1.000–1.001	0.808
TBIL	1.010	0.985–1.036	0.428
DBIL	0.967	0.905–1.034	0.326
IBIL	1.013	0.956–1.072	0.669
GGT	1.014	1.008–1.020	<0.001
HB	0.988	0.979–0.998	0.014
PLT	1.002	1.000–1.004	0.111
HBV			
HBV−			
OHB	2.328	1.495–3.623	<0.001
CHB	2.687	1.335–5.406	0.006

CHB: chronic hepatitis B (HBsAg+); OHB: occult hepatitis B (HBsAg−); AFP, alpha-fetoprotein; ALB, albumin; ALP, alkaline phosphatase; ALT, alanine aminotransferase; AST, aspartate aminotransferase; CA199, carbohydrate antigen 199; CEA, carcinoembryonic antigen; DBIL, direct bilirubin; GGT, γ-glutamyltransferase; HB, hemoglobin; IBIL, indirect bilirubin; PLT, platelet; TBIL, total bilirubin.

**Table 6:** Multivariate analysis for liver metastasis in the resectable group.

	Liver Metastasis		
	OR	95% CI	*p*
Age	1.013	0.985–1.041	0.387
Tumor size	2.176	1.615–2.932	0.028
T stage			0.054
T1–T2	1	1	
T3–T4	4.637	0.971–22.151	
N stage			0.908
N0–N1	1	1	
N2–N3	1.079	0.300–3.883	
Systematic treatment			0.650
No	1	1	
Yes	1.297	0.421–3.995	
HBV			0.017
HBV−	1	1	
HBV+	2.111	1.145–3.894	
CEA	1.000	1.000–1.000	0.540
CA199	1.000	1.000–1.000	0.425
GGT	1.012	1.005–1.020	0.001

CA199, carbohydrate antigen 199; CEA, carcinoembryonic antigen; GGT, γ-glutamyltransferase.

**Table 7:** Multivariate analysis for liver metastasis in the unresectable group.

	Liver Metastasis		
	OR	95% CI	*p*
Gender			0.007
Male	1	1	
Female	0.359	0.171–0.755	
Tumor size	2.205	1.655–2.973	0.028
HBV			0.003
HBV−	1	1	
HBV+	2.640	1.379–5.056	
AFP	1.002	1.001–1.003	0.005
CEA	1.000	1.000–1.000	0.305
ALT	1.023	0.988–1.059	0.199
AST	1.016	0.978–1.056	0.414
ALP	1.000	0.999–1.000	0.569
GGT	1.008	1.000–1.015	0.038

AFP, alpha-fetoprotein; ALP, alkaline phosphatase; ALT, alanine aminotransferase; AST, aspartate aminotransferase; CEA, carcinoembryonic antigen; GGT, γ-glutamyltransferase.

## Data Availability

The data presented in this study are available on request from the corresponding author due to institutional privacy regulations and patient confidentiality.

## Supplementary Materials

The following supporting information can be downloaded at https://www.mdpi.com/article/10.3390/curroncol33030179/s1, Table S1. Baseline characteristics; Table S2. Effect of hepatitis B virus (HBV) infection on liver metastasis in gastric cancer (GCLM) before and after propensity score matching (univariate analysis); Table S3. Univariate analysis for liver metastasis in the resectable group; Table S4. Univariate analysis for liver metastasis in the unresectable group; Figure S1. Effect of hepatitis B virus (HBV) infection on the overall survival (OS) of gastric cancer (GC) in the resectable group; Figure S2. Effect of hepatitis B virus (HBV) infection on the overall survival (OS) of gastric cancer (GC) in the unresectable group.

## Abbreviations

The following abbreviations are used in this manuscript:

HBV	Hepatitis B virus
GC	Gastric cancer
GCLM	Liver metastasis in gastric cancer
PSM	Propensity score matching
CHB	Chronic hepatitis B
OHB	Occult hepatitis B
OS	Overall survival
