# Supplementary material for: Hepatitis B Virus Infection Is Associated with a Higher Risk of Liver Metastasis in Gastric Cancer
Source: Curr Oncol. 2026 Mar 21;33(3):179. doi: 10.3390/curroncol33030179 (PMC13025660; doi:10.3390/curroncol33030179)
Supplement: Supplementary file 1 [file curroncol-33-00179-s001.zip › curroncol-4153084-supplementary.pdf]

Table S1. Baseline characteristics

Characteristic		
Gender		
Male		539(69.5)
Female		237(30.5)
Age, y		69.0(61.0, 74.0)
Primary GC		
Location		
Cardia		91(11.7)
Body		353(45.5)
Pylorus		332(42.8)
Tumor size, cm		3.6(2.8, 5.1)
Grade		
Poor		533(68.7)
Moderate/Well		243(31.3)
Resectability assessment		
No		259(33.4)
Yes		517(66.6)
Systematic treatment		
No		189(24.4)
Yes		587(75.6)
Neoadjuvant therapy		
No		595(76.7)
Yes		181(23.3)
Surgery		
No		199(25.6)
Yes		577(74.4)
Type		
Radical surgery with D2 lymphadenectomy		508(88.0)
Palliative surgery		60(10.4)
Endoscopic resection		9(1.6)
Number of lymph nodes resected		50(24, 39)
T stage		
T1-T2		197(25.4)
T3-T4		579(74.6)
N stage		
N0-N1		328(42.3)
N2-N3		448(57.7)
Liver cirrhosis		
No		754(97.2)
Yes		22(2.8)
HBV		
HBV-		
HBV+		
Liver metastasis		
No		625(80.5)
Yes	synchronous	109(14.0)
	metachronous	42(5.4)

Table S2. Effect of hepatitis B virus (HBV) infection on liver metastasis in gastric cancer (GCLM) before and after propensity score matching (univariate analysis).

	**Before PSM (*n* = 776)**			**After PSM (*n* = 600)**		
	**GCLM**			GCLM		
	**No (*n* = 625)**	**Yes (*n* = 151)**	p	**No (*n* = 478)**	**Yes (*n* = 122)**	*p*
Gender			0.010			0.108
Male	421(78.1)	118(21.9)		333(78.0)	94(22.0)	
Female	204(86.1)	33 (13.9)		145(83.8)	28(16.2)	
Age, y	68.0(60.5, 74.0)	69.0(63.0, 75.0)	0.147	67.5(60.0, 73.3)	69.0(62.0, 74.0)	0.265
Liver cirrhosis			0.484			0.216
No	606(80.1)	148(9.9)		465(80.0)	116(20.0)	
Yes	19(86.4)	3(13.7)		13(68.4)	6(31.6)	
Primary GC						
Location			0.429			0.674
Cardia	69(75.8)	22(24.2)		58(76.3)	18(23.7)	
Body	289(81.9)	64(18.1)		220(80.9)	52(19.1)	
Pylorus	267(80.4)	65(19.6)		200(79.4)	52(20.6)	
Tumor size, cm	3.6(2.4, 4.0)	5.1(5.1, 5.1)	<0.001	3.6(2.3, 4.0)	5.1(5.1, 5.1)	<0.001
Grade			<0.001			0.002
Poor	408(76.5)	125(23.5)		310(76.2)	97(23.8)	
Moderate/Well	217(88.2)	26(11.8)		168(87.0)	25(13.0)	
T stage			<0.001			<0.001
T1-T2	194(98.5)	3(1.5)		146(98.6)	2(1.4)	
T3-T4	431(74.4)	148(25.6)		332(73.5)	120(26.5)	
N stage			<0.001			<0.001
N0-N1	310(94.5)	18(5.5)		241(95.6)	11(4.4)	
N2-N3	315(70.3)	133(29.7)		237(68.1)	111(31.9)	
Systematic treatment			<0.001			0.597
No	175(92.6)	14(7.4)		106(78.5)	29(21.5)	
Yes	450(76.7)	137(23.3)		375(80.1)	93(19.9)	
HBV			0.001			0.002
HBV-	401(84.2)	75(15.8)		254(84.7)	46(15.3)	
HBV+	224(74.7)	76(25.3)		224(74.7)	76(25.3)	
AFP	2.7(2.0, 4.0)	3.1(2.2, 7.9)	<0.001	2.7(2.0, 3.9)	3.1(2.2, 8.2)	0.001
CEA	2.7(1.7, 5.1)	5.8(2.6, 31.0)	<0.001	2.8(1.7, 5.0)	5.4(2.6, 30.3)	<0.001
CA199	8.3(3.3, 38.8)	30.0(8.7, 351.2)	<0.001	8.6(3.3, 40.5)	31.6(7.4, 274.6)	<0.001
ALB	41.8(38.4, 44.5)	39.4(35.8, 42.0)	<0.001	41.9(38.6, 44.6)	39.4(35.9, 42.0)	<0.001
ALT	13.0(10.0, 19.0)	16.0(12.0, 27.0)	<0.001	13.0(10.0, 20.0)	16.5(12.0, 27.0)	<0.001
AST	19.0(15.0, 23.0)	23.0(17.0, 32.0)	<0.001	18.0(15.0, 23.0)	23.0(17.0, 32.0)	<0.001
ALP	73.0(58.0, 92.0)	92.0(66.0, 115.0)	<0.001	73.0(59.0, 91.3)	91.5(66.0, 115.0)	<0.001
TBIL	9.2(6.6, 12.7)	9.6(6.0, 14.1)	0.521	9.6(6.6, 12.7)	9.6(6.0, 14.2)	0.629
DBIL	3.3(2.4, 4.6)	3.7(2.3, 5.4)	0.096	3.3(2.4, 4.7)	3.7(2.3, 5.3)	0.187
IBIL	5.8(3.8, 8.4)	5.3(3.4, 8.2)	0.298	5.9(3.9, 8.4)	5.3(3.5, 8.4)	0.348
GGT	18.0(12.0, 27.0)	31.0(16.0, 83.0)	<0.001	17.0(12.0, 27.3)	31.0(16.8, 78.8)	<0.001
HB	128.0(108.5,142.0)	114.0(94.0, 128.0)	<0.001	129.0(109.0,143.0)	115.0(94.0, 128.3)	<0.001
PLT	234.0(185.0, 298.0)	260.0(196.0, 356.0)	0.003	235.0(185.0, 298.3)	260.5(191.8, 347.8)	0.016

GCLM: liver metastasis in gastric cancer; y, years; AFP, alpha-fetoprotein; ALB, albumin; ALP, alkaline phosphatase; ALT, alanine aminotransferase; AST, aspartate aminotransferase; CA199, carbohydrate antigen 199; CEA, carcinoembryonic antigen; DBIL, direct bilirubin; GGT, γ-glutamyltransferase; HB, hemoglobin; IBIL, indirect bilirubin; PLT, platelet; TBIL, total bilirubin

Table S3. Univariate analysis for liver metastasis in the resectable group.

**Characteristic**	**GCLM**		
	**No (*n* = 487)**	**Yes (*n* = 30)**	***p***
Gender			0.196
Male	335(93.3)	24(6.7)	
Female	152(96.2)	6(3.8)	
Age, y	68.0(61.0, 74.0)	71.5(69.0, 77.0)	0.048
Primary GC			
Location			0.584
Cardia	51(96.2)	2(3.8)	
Body	220(94.8)	12(5.2)	
Pylorus	216(93.1)	16(6.9)	
Tumor size, cm	3.5(2.0, 4.0)	5.0(3.5, 6.1)	<0.001
Grade			0.135
Poor	279(95.5)	13(4.5)	
Moderate/Well	208(92.4)	17(7.6)	
Systematic treatment			0.008
No	161(98.2)	3(1.8)	
Yes	326(92.4)	27(7.6)	
T stage			<0.001
T1-T2	189(99.0)	2(1.0)	
T3-T4	298(91.4)	28(8.6)	
N stage			0.008
N0-N1	297(96.4)	11(3.6)	
N2-N3	190(90.9)	19(9.1)	
HBV			<0.001
HBV-	307(97.5)	8(2.5)	
HBV+	180(89.1)	22(10.9)	
AFP	2.6(1.9, 3.9)	2.3(1.7, 3.4)	0.139
CEA	2.6(1.6, 4.5)	3.6(2.1, 7.8)	0.042
CA199	7.0(3.1, 20.3)	19.1(7.1, 53.3)	0.002
ALB	42.0(39.1, 44.7)	41.0(36.7, 42.7)	0.052
ALT	13.0(10.0, 20.0)	14.0(10.8, 20.0)	0.862
AST	19.0(15.0, 23.0)	19.0(16.0, 27.3)	0.454
ALP	73.0(58.0, 89.0)	65.0(46.5, 89.8)	0.075
TBIL	9.4(6.5, 12.7)	9.9(6.3, 14.2)	0.446
DBIL	3.3(2.4, 4.6)	3.8(2.2, 4.8)	0.784
IBIL	5.9(3.8, 8.4)	6.4(4.0, 9.6)	0.320
GGT	17.0(12.0, 26.0)	13.5(10.0, 21.5)	0.044
HB	130.0(112.0,144.0)	121.0(89.0, 140.3)	0.039
PLT	232.0(185.0, 291.0)	242.5(178.3, 298.8)	0.956

GCLM: liver metastasis in gastric cancer; y, years; AFP, alpha-fetoprotein; ALB, albumin; ALP, alkaline phosphatase; ALT, alanine aminotransferase; AST, aspartate aminotransferase; CA199, carbohydrate antigen 199; CEA, carcinoembryonic antigen; DBIL, direct bilirubin; GGT, γ-glutamyltransferase; HB, hemoglobin; IBIL, indirect bilirubin; PLT, platelet; TBIL, total bilirubin

Table S4. Univariate analysis for liver metastasis in the unresectable group.

**Characteristic**	**GCLM**		
	**No (n = 138)**	**Yes (n = 121)**	p
Gender			0.007
Male	86(47.8)	94(52.2)	
Female	52(65.8)	27(34.2)	
Age, y	69.0(59.8, 76.0)	69.0(62.0, 75.0)	0.793
Primary GC			
Location			0.491
Cardia	18(47.4)	20(52.6)	
Body	69(57.0)	52(43.0)	
Pylorus	51(51.0)	49(49.0)	
Tumor size, cm	3.6(3.6, 3.6)	5.1(5.1, 5.1)	<0.001
Grade			0.772
Poor	129(53.5)	112(46.5)	
Moderate/Well	9(50.0)	9(50.0)	
Systematic treatment			0.774
No	14(56.0)	11(44.0)	
Yes	124(53.0)	110(47.0)	
T stage			0.135
T1-T2	5(83.3)	1(16.7)	
T3-T4	133(52.6)	120(47.4)	
N stage			0.274
N0-N1	13(65.0)	7(35.0)	
N2-N3	125(52.3)	114(47.7)	
HBV			0.035
HBV-	94(58.4)	67(41.6)	
HBV+	44(44.9)	54(55.1)	
AFP	2.7(2.0, 4.0)	3.5(2.4, 11.6)	<0.001
CEA	3.9(2.0, 11.3)	6.9(2.9, 58.0)	0.001
CA199	34.4(5.2, 356.0)	47.8(10.6, 614.9)	0.234
ALB	40.2(36.9, 43.2)	38.7(35.6, 41.5)	0.058
ALT	11.0(9.0, 18.3)	18.0(12.0, 27.0)	<0.001
AST	18.5(15.0, 25.0)	23.0(17.5, 33.5)	<0.001
ALP	74.5(59.0, 110.3)	100.0(71.5, 132.5)	<0.001
TBIL	9.0(6.8, 12.4)	9.6(5.8, 14.2)	0.771
DBIL	3.2(2.5, 4.5)	3.7(2.3, 5.5)	0.282
IBIL	5.5(3.7, 8.3)	5.0(3.3, 7.8)	0.333
GGT	19.0(13.0, 33.3)	39.0(22.5, 116.5)	<0.001
HB	119.0(91.5,133.3)	112.0(94.0, 126.5)	0.120
PLT	248.0(184.0, 327.0)	267.0(201.6, 369.5)	0.055

GCLM: liver metastasis in gastric cancer; y, years; AFP, alpha-fetoprotein; ALB, albumin; ALP, alkaline phosphatase; ALT, alanine aminotransferase; AST, aspartate aminotransferase; CA199, carbohydrate antigen 199; CEA, carcinoembryonic antigen; DBIL, direct bilirubin; GGT, γ-glutamyltransferase; HB, hemoglobin; IBIL, indirect bilirubin; PLT, platelet; TBIL, total bilirubin

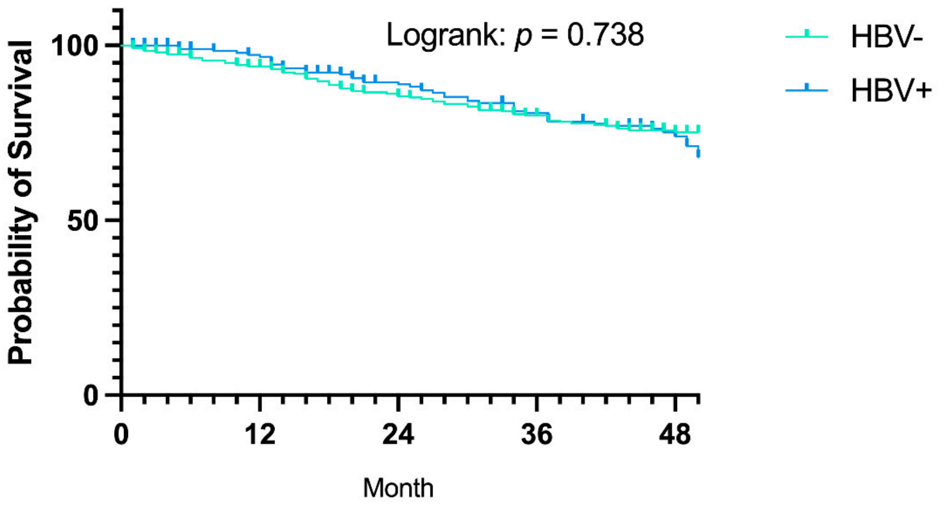

Figure S1. Effect of hepatitis B virus (HBV) infection on the overall survival (OS) of gastric cancer (GC) in the resectable group.

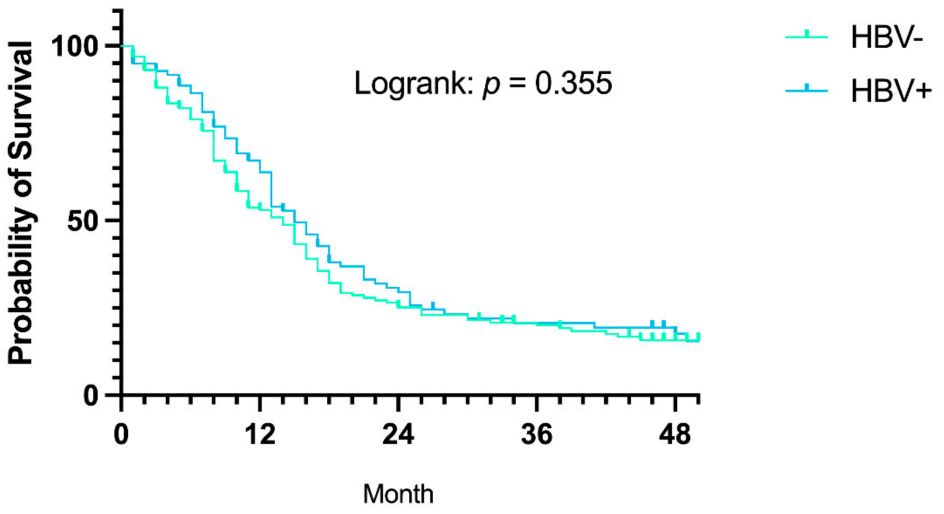

Figure S2. Effect of hepatitis B virus (HBV) infection on the overall survival (OS) of gastric cancer (GC) in the unresectable group.
